# Balance and gait adaptations in patients with early knee osteoarthritis^☆^

**DOI:** 10.1016/j.gaitpost.2014.01.005

**Published:** 2014-04

**Authors:** Lynsey D. Duffell, Dominic F.L. Southgate, Vivek Gulati, Alison H. McGregor

**Affiliations:** a MSK Lab, Imperial College London, UK; b Bioengineering Department, Imperial College London, UK

**Keywords:** Gait, Knee, Muscle, Osteoarthritis, Posture

## Abstract

Gait adaptations in people with severe knee osteoarthritis (OA) have been well documented, with increased knee adduction moments (KAM) the most commonly reported parameter. Neuromuscular adaptations have also been reported, including reduced postural control. However these adaptations may be the result of morphological changes in the joint, rather than the cause. This study aimed to determine if people with early OA have altered gait parameters and neuromuscular adaptations. Gait and postural tasks were performed by 18 people with early medial knee OA and 18 age and gender-matched control subjects. Parameters measured were kinematics and kinetics during gait and postural tasks, and centre of pressure and electromyographic activity during postural tasks. OA subjects showed no differences in the gait parameters measured, however they demonstrated postural deficits during one-leg standing on both their affected and unaffected sides and altered hip adduction moments compared with controls. Increased activity of the gluteus medius of both sides (*p* < 0.05), and quadriceps and hamstrings of the affected side (*p* < 0.05) during one-leg standing compared with controls were also noted. This study has demonstrated that gait adaptations commonly associated with OA do not occur in the early stages, while neuromuscular adaptations are evident. These results may be relevant for early interventions to delay or prevent osteoarthritis in its early stages.

## Highlights

• High knee adduction moments do not occur in early osteoarthritis.
• People with early knee-joint osteoarthritis show impairments in balance.
• Altered muscle activation is associated with early osteoarthritis during balance tasks.

## 1 Introduction

Despite knee osteoarthritis (OA) being the most common joint disorder in the UK, the pathogenesis of the disease is not fully understood. Mechanical adaptations in loading are thought to be a key factor in OA development and progression [1], and there is a body of evidence reporting kinematic gait adaptations in people with knee OA including reduced walking speeds, reduced knee flexion during weight acceptance and reduced overall range of motion at the knee joint [2–4]. Hip motion in the frontal plane has also been implicated in knee OA development, and one study reported that a greater hip abduction moment during gait protected against ipsilateral medial OA progression [5].

In terms of kinetics, the knee adduction moment (KAM), thought to represent loading on the medial compartment of the joint, is the most commonly reported parameter in medial knee OA. It is thought that an increased ground reaction force (GRF), combined with an increased lever arm distance between the knee joint centre and the GRF vector, due to malalignment, results in higher KAMs in OA subjects. Previous studies have reported an increased KAM during gait in people with medial knee OA [3,6,7], and this parameter has also been correlated with an increase in OA severity [4,8,9]. However, whether or not increased KAM is involved in the initiation of OA remains a topic of debate. Indeed some studies report no difference in KAM between OA and control subjects [10] and increased KAM has been correlated with a reduction in pain [11], indicating that higher KAMs are a consequence of pain reduction induced by pain relief medication.

Most studies to date have focussed on people during the latter stages of the condition and there is less evidence characterising gait adaptations early in the disease process. We recently reported that people with early OA show asymmetrical weight distribution during the sit-to-stand task in that they place more load through their unaffected side; in addition knee flexion and adduction moments in the affected side were not significantly different compared with control subjects [12]. Two recent review articles also concluded that there was limited evidence to suggest an involvement of KAM in early OA [2,9]. While there is evidence to suggest that increased KAM may be involved in the progression of OA, due to morphological changes in the joint [9], there is less evidence implicating higher KAM as a risk factor in the initial development of OA.

Neuromuscular adaptations are also relevant in knee OA; for example, reduced strength and proprioceptive accuracy of the quadriceps muscle [13,14]. People with knee OA have also been reported to have reduced balance, evidenced by a higher incidence of falls [15] and increased postural sway [16], as well as altered muscle activation patterns including increased activity and co-contraction of thigh muscles during the stance phase of gait [17,18]. Parameters related to motor control have however received little attention in people with early OA. The few studies that have been carried out report no differences in quadriceps strength [19] and proprioceptive accuracy at the knee joint [20] in people with early OA compared with control subjects. However, subtle differences in the neuromuscular responses during gait have been reported [18,21].

Further investigation is therefore relevant to clarify whether commonly reported adaptations in advanced OA are evident earlier in the disease process. Since it has been reported that early cartilage defects are reversible, particularly in younger people [22], identifying people with early OA, or with factors that put them at risk of OA, is extremely important to permit the possibility of reversing disease progression through early intervention. The aim of this study was to measure commonly reported gait parameters, associated with medial knee OA, in people with early medial knee OA and matched control subjects. In addition we measured static balance and muscle activation patterns during balance tasks in the same population. We hypothesised that people with early OA would have similar gait characteristics compared with controls but would have impaired balance.

## 2 Materials and methods

### 2.1 Subjects

This study had ethical approval from the South West London Research Ethics Committee and all subjects provided written informed consent.

OA Group: 18 subjects with clinical and radiographic evidence of unilateral early medial compartment knee OA (mild medial joint space narrowing, defined as 15–25% of joint space narrowing in the medial compartment of their diseased knee compared with the medial compartment of the healthy knee) were recruited from Charing Cross Hospital and local district regional hospitals. All subjects had presented to OA knee clinics with knee symptoms (swelling/stiffness and in some cases weakness/loss of confidence in their knee), however subjects reported no pain/swelling at the time of testing. Diagnosis was confirmed by a radiology hospital consultant and specialist orthopaedic registrar using radiographs and MRI scans. All subjects could ambulate without the use of an assistive device.

Control Group: 18 sex and age (±7 years) matched control subjects, with no clinical evidence of OA, were recruited from staff and students at Charing Cross Hospital and posters circulated in hospitals/gyms/local health centres.

Subjects were excluded from the study if they demonstrated any neurological or musculoskeletal condition other than knee OA, rheumatoid or other systemic inflammatory arthritis, morbid obesity (body mass index >35 km/m^2^) or had undergone previous surgical treatment for knee OA.

### 2.2 Motion capture protocol

Twenty reflective markers were positioned on the subject's pelvis and lower limbs and four marker clusters positioned on the subject's left and right thigh and calf segments. After cleaning the area with alcohol swabs, electromyography (EMG) electrodes were positioned on bilateral gluteus medius (GM), rectus femoris (RF), vastus lateralis (VL), vastus medialis (VM), biceps femoris (BF), semitendinosis (ST), soleus (S) and tibialis anterior (TA) muscles, according to SENIAM guidelines [23].

Two Kistler portable force plates (Kistler Type 9286B, Kistler Instrumente AG, Winterthur, Switzerland) were embedded into a 6 m walkway, and a 10 camera Vicon motion capture system was used to capture the position of the reflective markers (Vicon Motion Systems Ltd., Oxford, UK). A 16-channel wireless EMG system (Myon) was used to capture the activity of pelvis and lower limb muscles. The signals from the force plates and EMG were recorded using an analogue signal data acquisition card provided with the Vicon system and the Vicon Nexus software at a sampling rate of 1000 Hz. The motion capture was synchronised with the force and EMG data but was recorded at a rate of 100 Hz.

Subjects initially carried out maximal voluntary isometric contractions (MVICs) of all muscles measured three times, or until the EMG amplitude for the relevant muscle ceased to increase with a subsequent contraction. Subjects were then asked to walk at a comfortable speed along the 6 m walkway 5 times, or until three clean foot strikes had been recorded from each force plate. Subsequently, subjects were asked to stand on one force plate for 30 s, while staring at a fixed point on the wall and were requested to remain as still as possible for the duration of the task. This was repeated four times; (i) quiet standing with eyes open (QSEO); (ii) quiet standing with eyes closed (QSEC); (iii) one-leg standing on left leg (OLS-L) and; (iv) one-leg standing on right leg (OLS-R).

### 2.3 Data analysis

For gait and OLS-L and OLS_R data, kinematic and kinetic parameters in the sagittal and coronal planes at the hip and knee joints were calculated from the position of the reflective markers and the ground reaction force data using a custom model written in body builder software [24,25]. Joint moments were normalised to the subject's bodyweight × height. Data were separated into gait cycles (stance phase only) for each leg based on force plate vertical ground reaction forces (≥40 N = heel strike, ≤40 N = toe-off). For gait data, discrete parameters were measured as follows; knee angle in the sagittal plane (i) at initial contact, (ii) at peak knee flexion, and (iii) range of motion; hip angle in the frontal plane (i) at initial contact; knee moment in the frontal plane (i) at 1st peak, and (ii) mean during stance; and hip moment in the frontal plane (i) at 1st peak, and (ii) mean during stance. For OLS data, an average of the central 20-s period was taken.

EMG data were band pass filtered (10–200 Hz), full wave rectified and low-pass filtered (2nd order, 6 Hz cut-off). EMG data recorded during the balance tasks (QSEO, QSEC, OLS-L and OLS-R) were then normalised to the activity of the relevant muscle during the MVIC and an average of the central 20-s period for each balance task. Additionally, centre of pressure (COP) trajectory distance was measured from force plate data (COP position in the medio-lateral and antero-posterior directions) during the central 20-s period for each balance task and expressed relative to time (mm/s); all postural tasks were completed with the same amount of time.

Statistical analysis was carried out using SigmaPlot for Windows (v. 11.0). Comparisons were made between control (right and left sides) and OA subjects (affected and unaffected sides). All data was tested for normality using the Shapiro-Wilk test, and Levene's test was used to test for equality of variance. For kinematic and kinetic data during gait and OLS, two-way Analysis of Variance (ANOVA) was used (group × leg) to assess differences. Significance was set at *p* < 0.05. ANOVA's were also used to assess COP and EMG data for each muscle across balance tasks (QSEO, QSEC and OLS).

## 3 Results

**Fig. 1:**
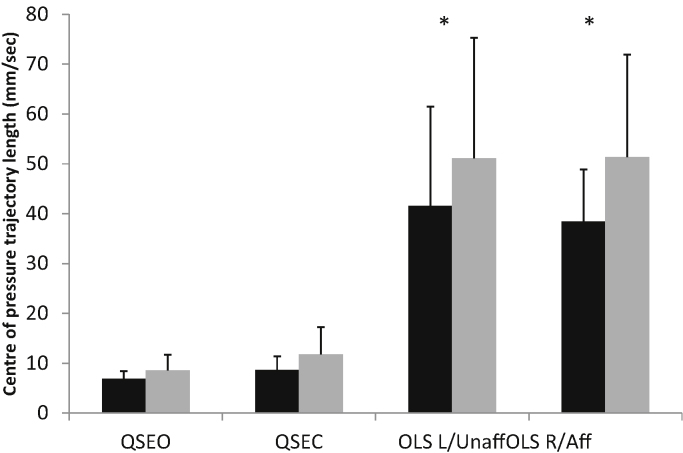
Mean (SD) centre of trajectory path length for controls (black) and early OA subjects (grey) during quiet standing with eyes open (QSEO) and eyes closed (QSEC) and one leg standing on the left (controls) or unaffected (OA) side (OLS L/Unaff) and right (controls) or affected (OA) side (OLS R/Aff). * denotes significant difference between groups (*p* < 0.05).

**Fig. 2:**
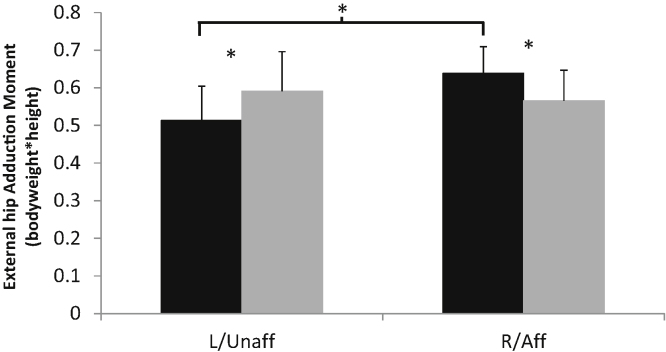
Mean (SD) hip adduction moment for controls (black) and early OA subjects (grey) during one leg standing on the left (controls) or unaffected (OA) side (L/Unaff) and right (controls) or affected (OA) side (R/Aff). * denotes significant difference between groups (*p* < 0.05).

**Fig. 3:**
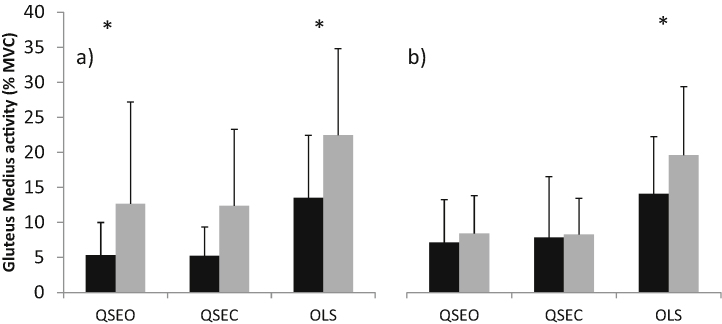
Mean (SD) activity of the gluteus medius muscle for controls (black) and early OA subjects (grey) of the (a) left (controls) or unaffected (OA) side and (b) right (controls) or affected (OA) side for quiet standing with eyes open (QSEO) and eyes closed (QSEC) and one leg standing (OLS). * denotes significant difference between groups (*p* < 0.05).

Control and OA subjects had a mean (SD) age of 56.2 (13.0) and 56.4 (12.4) years, height of 1.68 (0.09) and 1.68 (0.11) m, and weight of 68.1 (13.7) and 75.1 (12.5) kg, respectively. Subjects were similar in age and height, however OA subjects were significantly heavier (*p* < 0.05). There were no significant differences between the control and OA subjects for all kinematic and kinetic parameters measured during gait (Table 1). During the balance tasks, COP trajectory length was similar between groups during QSEO and QSEC, it was however significantly longer for OA subjects during OLS both on affected and unaffected sides (*p* < 0.05; Fig. 1). There were no significant differences in kinematic data during OLS. Control subjects had significantly higher hip adduction moments (HAM) on their right compared with left sides (*p* < 0.05), and OA subjects had significantly higher HAMs on their unaffected side compared with controls left side (*p* < 0.05), and significantly lower HAMs on their affected side compared with controls right side (*p* < 0.05; Fig. 2). No other significant findings were noted in the kinetic data. EMG data showed that during one-leg standing OA subjects had significantly greater activation of the gluteus medius muscle (ipsi-lateral to the side on which they were standing) for both the affected and unaffected sides (*p* < 0.05; Fig. 3a and b). GM on the unaffected side also showed greater activation during QSEO (*p* < 0.05; Fig. 3a). In addition, OA subjects had greater activation of quadriceps (VL and VM) and Hamstrings (BF) muscles during OLS on the affected side only (*p* < 0.05). There were no significant differences in the EMG activity of all other muscles measured.

## 4 Discussion

We aimed to investigate parameters commonly associated with early knee joint OA, specifically related to gait and neuromuscular parameters. We have noted no differences in gait parameters in people with early medial knee-joint OA and matched control subjects, however people with early OA were found to have increased postural sway during one-leg standing, which may be related to the altered muscle activation patterns noted during these tasks.

The knee adduction moment (KAM) is a commonly reported parameter in people with knee OA. While there is extensive evidence to support higher KAMs in people with severe knee OA [3,6,7], and evidence to suggest KAM correlates with OA severity [4,8,9], there is limited evidence to suggest this occurs in early OA, indicating it may not be a risk factor [9]. Our data support this concept in people with early OA, as we noted no significant differences in either the 1st peak or mean KAM during gait in subjects with early OA compared with controls. Subchondral bone expansion has been proposed to be the primary event in knee OA [26] and we recently reported a correlation between the size of the tibial plateau measured from 3D computed tomography scans, and KAM measured during gait using 3D motion capture, in people with severe medial knee joint OA [27]. Therefore, commonly reported increases in KAM may only occur in more severe cases, due to morphological changes in the pathological joint [9]. Increased KAM has also been correlated with a reduction in pain [11] thus it may also be associated with the use of pain relief medication.

Reduced flexion during stance has also been noted in people with knee OA [3,4] and implicated in its development because reduced knee flexion during weight acceptance is thought to reduce the amount of energy normally absorbed through knee flexion [17], thereby increasing forces. Our data indicate that this process does not occur in the early stages of OA and this may also be related to structural changes in the joint or a strategy to avoid pain in this population. Other relevant gait parameters are associated with the hip joint, and a greater hip abduction moment during gait has been suggested to be protective against ipsilateral medial OA progression longitudinally [5]. Our data do not support the role of the kinematics or kinetics in the frontal plane of the hip joint in the early development of knee OA.

Neuromuscular changes are known to occur with normal ageing, with a higher incidence of falls reported in older subjects [28]. However, the development of OA is associated with additional neuromuscular deficits and a higher incidence of falls [15] and greater postural sway [16] has been noted in people with OA compared with control subjects of similar age. There is limited work investigating neuromuscular deficits in the early stages of OA development; our data indicate that alterations in muscle activation patterns and postural sway do occur in these subjects when compared with age- and gender-matched control subjects. Specifically, OA subjects had increased postural sway when standing on one-leg on both their affected and unaffected sides. They also demonstrated increased activation of the quadriceps and hamstrings muscles when standing on their affected side only, and increased gluteus medius activity, a hip abductor muscle, during quiet standing and one-leg standing on both their affected and unaffected sides. Kinetic outputs during OLS indicated that there were differences between control and OA subjects. The increased external hip adduction moment on the unaffected side of OA compared with control subjects may explain the concurrent increased gluteus medius activity during that task. This seems unlikely however because, on their affected side, OA subjects had lower HAM compared with controls but the gluteus medius activity was again increased. It is interesting to note that control subjects had significantly higher HAMs on their right compared with left sides. This indicates the leg dominance may be relevant, and therefore this factor should be considered in future studies.

It has been proposed that pain causes reduced postural control, since experimentally induced pain impairs postural stability in healthy individuals [29]. However, this does not explain our data since the subjects reported no pain in their affected side (on the day of testing) and reduced postural control was also noted when subjects were standing on their unaffected limb. Muscle weakness and reduced proprioception have also been related to alterations in postural stability [30]. While these parameters were beyond the scope of the present study, previous studies have indicated that both quadriceps strength [19] and proprioceptive accuracy [20] are similar in people with early OA compared with control subjects. It is possible that the altered muscle activity observed in this study, notably in the gluteus medius muscle on both the affected and unaffected sides, is related to the reduction in postural control, either as a causative or compensatory factor.

The increased activation of the vastus lateralis, vastus medialis and biceps femoris muscles during one-leg standing on the affected side of subjects with early OA is similar to that commonly reported in OA subjects during the stance phase of gait [17,18], and also previously noted in people with early OA [18]. This may be a protective mechanism over the knee joint, which occurs early in the disease process or may be a risk factor for knee OA.

It should be acknowledged that the groups in this study were not matched in terms of body mass and the OA subjects were significantly heavier compared with controls; all force data were therefore body-weight normalised. Subjects were also not matched for other factors associated with the development of knee OA; while this would be ideal, it is unrealistic to control for such a wide range of factors. Our data indicate that few gait adaptations can be noted in people with early knee OA, however neuromuscular alterations are evident. Thus, in terms of understanding the causes of OA, more attention should be given to neuromuscular adaptations that occur early in the disease process and these should be measured longitudinally in people at high risk of OA development. Since neuromuscular adaptations are easily targeted by therapy, and it has been suggested that early cartilage defects are reversible [22], such therapeutic interventions require further attention to prevent OA or to delay or reverse OA progression.

In conclusion, we have noted no differences in commonly reported kinematic and kinetic variables measured during gait in people with early OA. People with early medial knee joint OA did however have reduced postural stability on both their affected and unaffected limbs and altered muscle activation patterns, specifically related to gluteus medius and the quadriceps and hamstrings. Further work is required investigating the neuromuscular adaptations associated with early OA to improve our understanding of the causative factors in OA development and to inform early rehabilitation strategies.

## Figures and Tables

**Table 1:** Mean (SD) kinematic and kinetic discrete parameters during gait for control and OA groups.

		Control		OA	
		Left	Right	Unaffected	Affected
Knee flexion angle (degrees)	At initial contact	5.9 (4.6)	6.9 (4.6)	7.2 (4.2)	4.4 (8.3)
	Peak	18.5 (7.0)	18.4 (5.9)	18.8 (6.0)	14.2 (10.0)
	Range of motion	16.0 (5.7)	16.4 (4.4)	14. 8 (5.6)	12.5 (4.9)
Hip adduction angle (degrees)	At initial contact	−2.8 (5.5)	−0.3 (4.2)	−2.0 (2.9)	−2.4 (3.7)
External knee adduction moment (Nm/weight*height)	1st peak	0.21 (0.07)	0.23 (0.13)	0.19 (0.05)	0.21 (0.08)
	Mean	0.13 (0.06)	0.13 (0.08)	0.12 (0.05)	0.12 (0.10)
External hip adduction moment (Nm/weight*height)	1st peak	0.51 (0.10)	0.56 (0.12)	0.51 (0.09)	0.55 (0.14)
	Mean	0.35 (0.07)	0.39 (0.10)	0.37 (0.06)	0.38 (0.08)
